# Selective Enhancement of Donor Hematopoietic Cell Engraftment by the CXCR4 Antagonist AMD3100 in a Mouse Transplantation Model

**DOI:** 10.1371/journal.pone.0011316

**Published:** 2010-06-28

**Authors:** Yubin Kang, Benny J. Chen, Divino DeOliveira, Jeffrey Mito, Nelson J. Chao

**Affiliations:** 1 Divisions of Hematology, Oncology and Cellular Therapy, Department of Medicine, Duke University Medical Center, Durham, North Carolina, United States of America; 2 Division of Cellular Therapy/Adult Bone Marrow Transplantation, Department of Medicine, Duke University Medical Center, Durham, North Carolina, United States of America; Harvard Medical School, United States of America

## Abstract

The interaction between stromal cell-derived factor-1 (SDF-1) with CXCR4 chemokine receptors plays an important role in hematopoiesis following hematopoietic stem cell transplantation. We examined the efficacy of post transplant administration of a specific CXCR4 antagonist (AMD3100) in improving animal survival and in enhancing donor hematopoietic cell engraftment using a congeneic mouse transplantation model. AMD3100 was administered subcutaneously at 5 mg/kg body weight 3 times a week beginning at day +2 post-transplant. Post-transplant administration of AMD3100 significantly improves animal survival. AMD3100 reduces pro-inflammatory cytokine/chemokine production. Furthermore, post transplant administration of AMD3100 selectively enhances donor cell engraftment and promotes recovery of all donor cell lineages (myeloid cells, T and B lymphocytes, erythrocytes and platelets). This enhancement results from a combined effect of increased marrow niche availability and greater cell division induced by AMD3100. Our studies shed new lights into the biological roles of SDF-1/CXCR4 interaction in hematopoietic stem cell engraftment following transplantation and in transplant-related mortality. Our results indicate that AMD3100 provides a novel approach for enhancing hematological recovery following transplantation, and will likely benefit patients undergoing transplantation.

## Introduction

Hematopoietic cell transplantation (HCT) provides a potentially curative treatment approach for a variety of diseases[1]. However, HCT is associated with high incidence of morbidity and mortality. Reducing transplant-related mortality and facilitating donor cell engraftment are very important goals in HCT. Recombinant granulocyte colony stimulating factor (G-CSF) is commonly used to accelerate neutrophil recovery following HCT[2]; its effects are limited to myeloid cells[3].

Hematopoietic stem cell (HSC) niche is a specific site in the marrow cavity where HSCs reside and undergo self-renewal, proliferation and differentiation[4]. HSC niche consists of supporting cells such as osteoblasts and other stromal cells that provide a microenvironment for HSCs, as well as molecules produced by these cells. At least two types of HSC niches have been identified in the bone marrow: osteoblastic/endosteal niche and vascular endothelial niche[5], [6]. The osteoblastic/endosteal niche is thought to be the primary site for very primitive, quiescent HSCs; while the vascular endothelial niche is the predominant site for activated HSCs that are on their way to be released into circulation. The detailed structure and the roles of these 2 niches in hematopoiesis remain to be elucidated[7].

Following HCT, donor HSCs home to the marrow niches (mainly osteoblastic niche), engraft, proliferate, and eventually reconstitute the whole hematological and immunological repertoire of the recipient. HSC engraftment is limited by the availability of niches during and after transplant when HSCs still survive. Increasing the number of “unoccupied” marrow niches enhances transplant efficiency[8], [9], [10]. The proliferative status of HSCs also affects their “transplantability” and engraftment. Some studies suggest that dividing HSCs are more efficient than resting HSCs in engraft[11] and in vivo proliferation of HSCs is crucial to hematological recovery following transplantation[12], [13], while others found that proliferating HSCs displayed engraftment defect when transiting S/G_2_/M phase[14], [15].

An array of chemokines, growth factors, cell-surface and adhesion molecules are required to function in concert for HSCs to home to the niches and to engraft following HCT. In particular, the interaction of stromal cell-derived factor-1 (SDF-1, CXCL-12) in the marrow matrix with the CXCR4 chemokine receptor on HSCs plays a critical role in all aspects of donor cell reconstitution including HSC homing[16], [17], [18], engraftment[9] and proliferation[14], [19], [20]. SDF-1 is secreted by endothelial cells, osteoblasts and other stromal cells, and is present in marrow extracellular matrix. CXCR4 is expressed on HSCs, osteoblasts, T cells and other inflammatory cells. SDF-1 is the only known ligand for the CXCR4 receptor. Following transplantation conditioning, the level of SDF-1 in the marrow microenvironment significantly increases. The gradient difference of SDF-1 between the niche microenvironment and blood stream promotes HSC transendothelial migration and homing to the niches[17]. Additionally, SDF-1 in the marrow extracellular matrix binds to the CXCR4 receptors on HSCs and has dual effects: tethering HSCs in the niches, and arresting the cycling of very primitive HSCs[14], [19], [20].

AMD3100 is a highly specific and reversible antagonist of the CXCR4 receptor[21]. AMD3100 is a bicyclam molecule with an aromatic bridge[21], and binds with extreme specificity to CXCR4, with contacts at amino acid positions 171, 182, 193 and 262 of CXCR4. AMD3100 was originally studied as an agent for the treatment of patients infected with human immunodeficiency virus (HIV) since some strains of HIV require CXCR4 as a co-receptor for cell entry. However, during initial clinical trials, rapid and reversible leukocytosis was noted in human volunteers and in HIV patients treated with AMD3100 and led the focus of AMD3100 development towards hematopoietic stem cell mobilization[22], [23]. AMD3100 blockade of the SDF-1/CXCR4 interaction results in rapid release of HSCs from marrow niches and mobilization of HSCs into the peripheral blood[17], [24], [25], [26], [27], [28]. AMD3100 was recently approved by the Food and Drug Administration (FDA) to be used in combination with G-CSF for stem cell mobilization in patients with non-Hodgkin's lymphoma and multiple myeloma.

Most of the work with AMD3100 has been exclusively on its use as a mobilization agent[24], [27], [28], [29], [30]. We hypothesized that post transplant administration of AMD3100 would enhance donor cell reconstitution in myeloablative recipients. We reasoned that AMD3100 would induce *in vivo* proliferation of transplanted donor stem cells and increase additional availability of marrow niches for donor stem cell engraftment. We report here the effects of post transplant administration of AMD3100 on animal survival and on donor cell engraftment in a mouse transplantation model.

## Results

### 1. Post transplant administration of AMD3100 significantly improves animal survival

**Figure 1:**
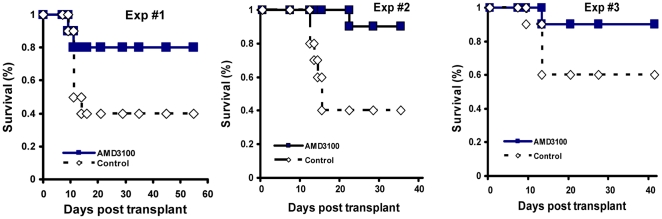
Post transplant administration of AMD3100 improves animal survival in a murine transplantation model. KTLS cells from C57BL/Ka CD45.1 Thy1.1 mice were injected via tail vein into lethally irradiated C57BL/6 CD45.2 Thy1.2 mice (250 KTLS cells per mouse). Beginning 2 days after transplant (day +2), the mice were injected subcutaneously with PBS control or AMD3100 at 5 mg/kg body weight every Monday, Wednesday, and Friday until day +56. Animal survival was monitored daily. In experiment #1 and #2, the mice received 10.5 Gy total body irradiation using a Cesium irradiator. In experiment #3, the mice received 9.5 Gy total body irradiation. (n = 10 mice in each group in each experiment).

We first examined the effects of post transplant administration of AMD3100 on animal survival following transplantation. We injected sorted c-Kit^+^ Thy1^low^ Lin^−^ Sca-1^+^ hematopoietic stem cells (KTLS cells) from C57BL/Ka **CD45.1** Thy1.1 mice via tail vein into lethally irradiated C57BL/6 **CD45.2** Thy1.2 mice (250 KTLS cells/recipient mouse). Transplanted mice were then injected subcutaneously with PBS control buffer or AMD3100 (5 mg/kg body weight) every Monday, Wednesday and Friday beginning at day +2 post transplant for a total of 56 days. Animal survival was monitored daily. We performed 3 separate sets of experiments with 10 mice per group in each experiment. The mice received 9.5–10.5 Gy total body irradiation. At the 10.5 Gy dose, 60% of transplanted mice receiving PBS control died within 2 weeks following transplantation. In contrast, only 13.3±3% (mean ± SEM) of transplanted mice treated with AMD3100 died. The animal survival results from our 3 separate sets of experiments are shown in **Fig. 1**. These data indicate that post transplant administration of a CXCR4 antagonist (AMD3100) significantly improves animal survival following HCT.

### 2. Post transplant administration of AMD3100 selectively enhances donor cell recovery

**Figure 2:**
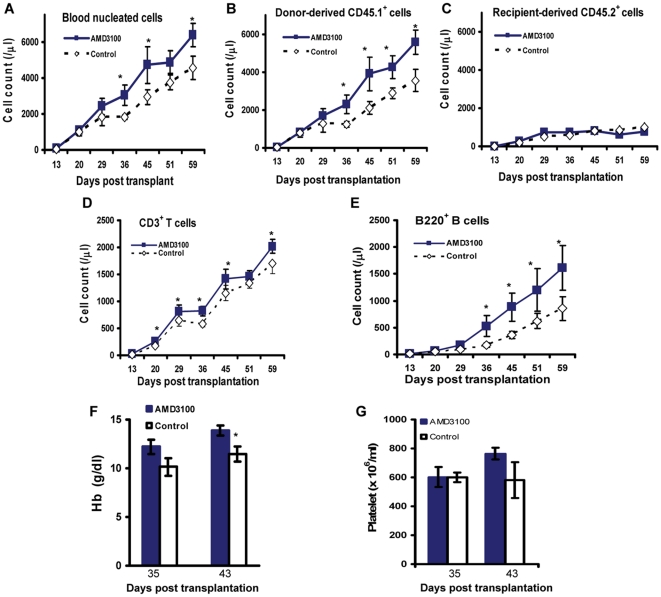
Post transplant administration of AMD3100 selectively enhances reconstitution of all donor-derived hematological lineages of cells. KTLS cells from C57BL/Ka CD45.1 Thy1.1 mice were injected via tail vein into lethally irradiated C57BL/6 CD45.2 Thy1.2 mice (250 KTLS cells per mouse). Beginning 2 days after transplant (day +2), the mice were injected subcutaneously with PBS control or AMD3100 at 5 mg/kg body weight every Monday, Wednesday, and Friday until day +56. Hematological recovery and cell subset analyses were determined at the time points indicated, as described in Methods. The graphs are representative of 3 separate sets of experiments with 10 mice in each group in each experiment. A: Blood nucleated cell counts (/µl) (normal range of mouse white cell counts: 1.8–10.7×10^3^/µl). B: Donor-derived CD45.1^+^ cell counts (/µl). C: Blood recipient origin CD45.2^+^ cell counts (/µl). D: Blood CD3^+^ T cell counts (/µl). E: Blood B220^+^ B cell counts (/µl). F: Hemoglobin concentration (g/dl) (normal range of mouse hemoglobin level: 11–15.1 g/dl). G: Blood platelet counts (×10^6^/ml) (normal range of mouse platelet count: 592–2972×10^6^/ml). *: p<0.05

To determine the effects of AMD3100 on hematological recovery, we performed blood counts weekly beginning at Day +7. The total nucleated cells, the donor-derived cells (CD45.1^+^ cells), and the recipient-origin cells (CD45.2^+^ cells) in the peripheral blood were measured using multi-color flow cytometry analyses. As demonstrated in **Fig. 2A**, mice given AMD3100 had faster nucleated cell recovery. The difference in blood nucleated cell counts between AMD3100-treated mice and PBS-injected control mice became statistically significant beginning at Day +36, and was completely attributed to faster donor-derived cell recovery (**Fig. 2B**). Contribution of recipient-derived cells (CD45.2^+^) was minimal (<10%) in all transplanted mice and there was no difference in the number of recipient-derived CD45.2^+^ cells between AMD3100 injected mice and mock control mice (**Fig. 2C**). These data demonstrate that AMD3100 selectively augments donor derived cell reconstitution.

We next performed multi-color cell subset analyses on the blood samples. We found that AMD3100 injection augmented the reconstitution of not only neutrophils, but also B cells and T cells (**Fig. 2D and 2E**). Again, the difference was predominantly attributed to enhanced recovery of donor derived cells (data not shown). In a separate set of experiment, we measured the hemoglobin concentration and platelet count at the indicated time-points. As shown in **Fig. 2F**, AMD3100 administration significantly enhanced red blood cell recovery. Although the platelet count was higher in AMD3100-treated mice at day +43 post transplantation, the difference was not statistically significant (**Fig. 2G**). This may be because the platelet count had reached normal levels in both groups at the time the platelet counts were measured. Nevertheless, these data suggest that AMD3100 administration enhances reconstitution of all hematological lineages.

### 3. Post transplant administration of AMD3100 reduces pro-inflammatory cytokine production

**Figure 3:**
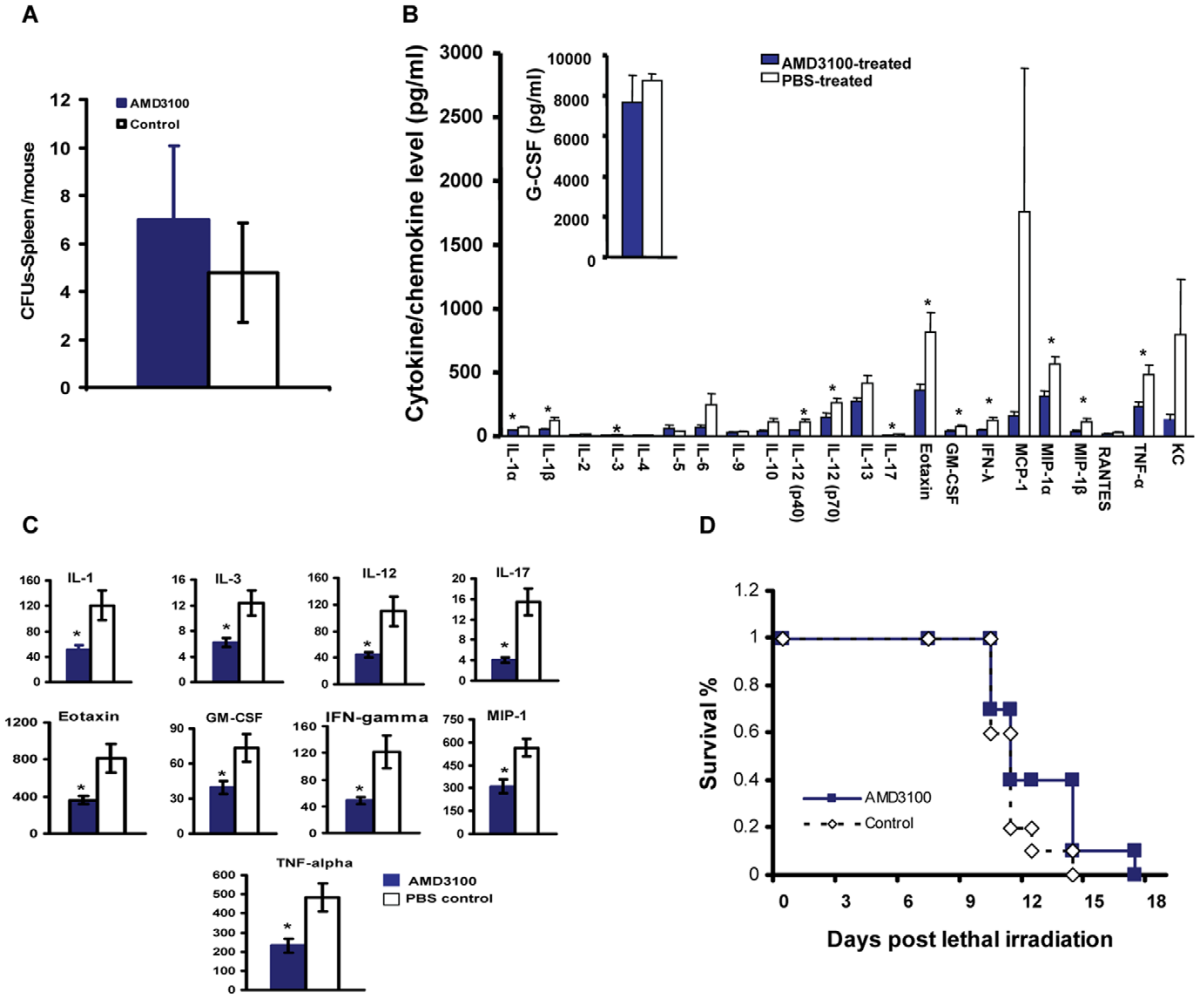
Post transplant administration of AMD3100 increases CFUs-spleen and reduces cytokine, chemokine, and growth factor levels. A: CFUs-spleen assay. Lethally irradiated CD45.2 mice (10.5 Gy) were injected with whole marrow cells from CD45.1 mice (1×10^5^ marrow cells per mouse). The recipient mice were injected subcutaneously with PBS buffer or AMD3100 at 5 mg/kg every other day beginning at day +2. At day +9, the mice were sacrificed and CFUs-spleen measured (n = 5–10 mice per group). B: Plasma cytokine/chemokine measurement. Lethally irradiated CD45.2 mice (10.5 Gy) were injected with sorted KTLS stem cell from CD45.1 mice (7000 KTLS cells per mouse). The recipient mice were injected subcutaneously with PBS buffer or AMD3100 at 5 mg/kg every other day beginning at day +2. The mice were bled at Day +7 and plasma prepared. Plasma cytokines and chemokines (pg/ml) were measured as per manufacturer's instruction (Bioplex, Bio-Rad Laboratories) (n = 5 mice per group). G-CSF level was shown in a separate panel. C: 9 cytokines/chemokines that were significantly reduced in AMD3100-treated mice. D: Survival rate in lethally irradiated mice without HCT. CD45.2 mice were lethally irradiated (10.5 Gy) but not transplanted with HSCs. The mice were then administered subcutaneously with PBS or AMD3100 at 5 mg/kg every other day beginning at day +2 post irradiation until the end of experiments. Animal survival was monitored daily (data representative of 3 separate sets of experiments with 10 mice in each group of each experiment).

It is noteworthy that there is a temporal difference between animal survival benefit and the blood hematological recovery seen in AMD3100-treated mice. The reduced mortality seen in AMD3100-treated mice occurred mainly within 2 weeks, a period before any blood cell count differences were apparent between AMD3100-treated and PBS-injected mice. One possible explanation for this time gap could be that AMD3100 promotes earlier hematopoietic progenitor recovery. To determine whether post transplant administration of AMD3100 would affect hematopoietic progenitor cell recovery, we measured CFUs-spleen at day +9. As shown in **Fig. 3A**, AMD3100-treated mice demonstrated a trend to higher number of CFUs-spleen compared to PBS- injected control mice.

Another possible mechanism may be related to AMD3100 mediated- inhibition of a cytokine storm. Transplant preparative conditioning induces massive cell death and tissue damage, resulting in a dramatic increase in the levels of secreted chemokines, cytokines and proteolytic enzymes[16]. This massive release of cytokines/chemokines (a “cytokine storm”) exacerbates inflammation and vascular/tissue injuries, and leads to multi-organ toxicities. CXCR4 plays an important role in the cytokine storm by recruiting inflammatory cells to the injury sites[31], [32]. We reasoned that post transplant administration of AMD3100 would attenuate the cytokine storm, and hence reduce multi-organ toxicities. We measured a total of 23 serum cytokines at Day +7 post transplantation (**Fig.3B**). We found that post transplant administration of AMD3100 led to a significant reduction in the levels of 9 pro-inflammatory cytokines/chemokines (IL-1, IL-3, IL-12, IL-17, tissue necrosis factor-α, GM-CSF, interferon-λ, macrophage-inhibitory protein-1 and eotaxin) (**Fig. 3C**).

We next examined if AMD3100 could protect mice from lethal irradiation without the need for HCT. Lethally irradiated C57BL/6 **CD45.2** Thy1.2 mice (10.5 Gy) were given AMD3100 at 5 mg/kg body weight either at day 2 post irradiation and then every other day until the death of mice or within one hour post irradiation and then daily for 5 days. As shown in **Fig. 3D**, without HSC transplant, all the irradiated mice died by day 17 and there were no significant differences in animal survival between AMD3100-treated mice and PBS-injected mice. These data imply that the effects of AMD3100 on HSCs may indeed play a critical role in improving animal survival.

### 4. Post transplant administration of AMD3100 enhances donor marrow cell engraftment

**Figure 4:**
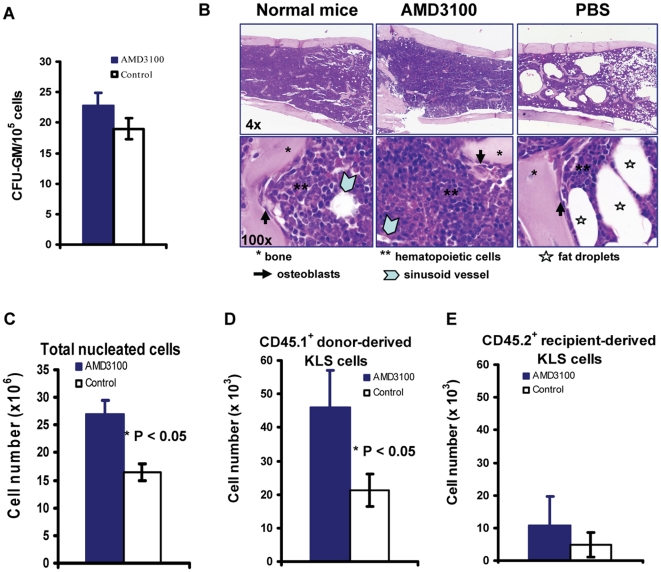
Post transplant administration of AMD3100 enhances donor cell marrow engraftment. The mice as described in **Fig. 1** were sacrificed at Day +65 post transplantation, and 2 femurs and 2 tibias were harvested and processed and analyzed as described in Methods. A: CFU-GM assay. CFU-GM colonies were measured in individual mice using MethoCult® GF 3434 mouse colony-forming cell assay. B: Histology. Bone marrow from individual mouse was examined by microscopy (4X: top panel and 100X: bottom panel) after decalcification and H/E staining. Trabecular bony structure, osteoblasts, hematopoietic cells, sinusoid vessels and acellular areas filled with lipid droplets were indicated. Normal mice did not receive transplant or AMD3100 injection. C: Total nucleated cell number from 1 femur and 1 tibia. D: Donor-derived CD45.1^+^ KLS stem cell number from 1 femur and 1 tibia of individual mouse. E: Recipient-origin CD45.2^+^ KLS stem cell number from 1 femur and 1 tibia of individual mouse. *: p<0.05.

To further investigate the effects of AMD3100 on marrow hematopoietic progenitor/stem cell recovery, we administered the last AMD3100 injection at day +56 and sacrificed the mice at day +65 and harvested bone marrow for CFU-GM assay, histopathological examination and flow cytometry analyses. Compared to PBS-injected- mock control mice, mice receiving AMD3100 injection had increased frequencies of CFUs-GM (represented as colony numbers/10^5^ marrow nucleated cells, **Fig. 4A**) and had a much denser and more cellular marrow (**Fig. 4B**). While the trabecular bony structures, osteoblasts and sinusoid vessels were preserved, the hematopoietic cell components were significantly expanded in AMD3100-treated mice. Furthermore, 3 of 4 PBS-injected- mock control mice had large areas filled with lipid droplets and devoid of cells (**Fig. 4B**, indicated as stars). These large acellular areas are commonly associated with radiation[33]. In contrast, the large areas (fat droplets) were not found in any of the mice receiving AMD3100 (8 of 8 mice) (**Fig. 4B**). In addition, we collected one femur and one tibia from each mouse and measured total nucleated marrow cells, total donor-derived CD45.1^+^ KLS hematopoietic stem cells (c-kit^+^ Lin^−^ Sca-1^+^ cells) and recipient-origin CD45.2^+^ KLS cells. As shown in **Fig. 4C** and **4D**, AMD3100-treated mice had significantly higher numbers of total marrow nucleated cells and total donor-derived CD45.1^+^ KLS cells per one femur and one tibia (One femur plus one tibia account for ∼7.5% of whole body marrow cells[34]). There was no difference in the recipient- derived CD45.2^+^ KLS cell (**Fig. 4E**). These data again suggest that post transplant administration of AMD3100 selectively enhances donor cell marrow engraftment. The total nucleated cells, the donor-derived CD45.1^+^ KLS cells, and the recipient-origin CD45.2^+^ KLS cells in the spleen were not significantly different between AMD3100-treated mice and PBS-injected mock mice (data not shown).

### 5. Post transplant administration of AMD3100 mobilizes recipient's residual stem cells, increasing marrow niche availability

**Figure 5:**
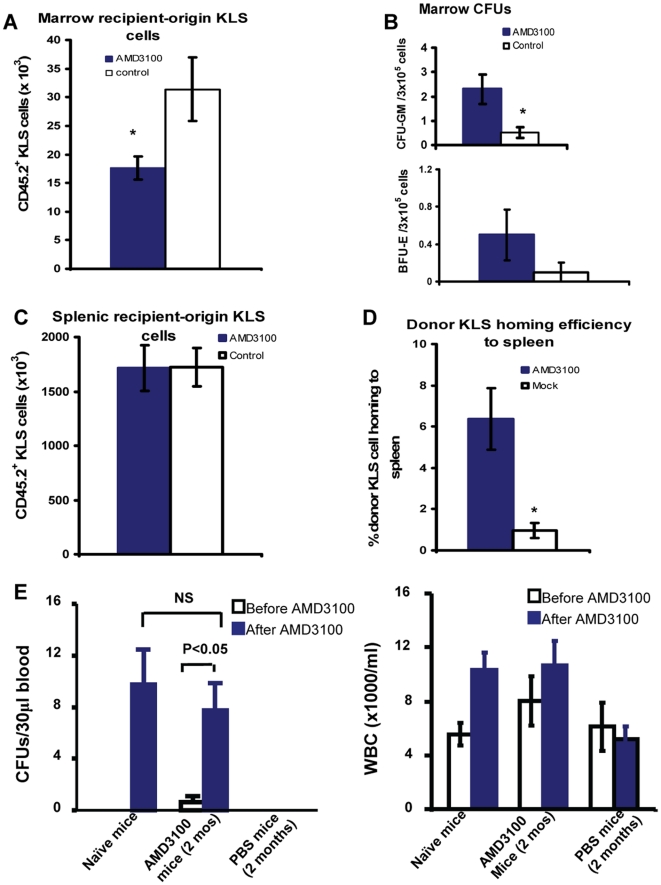
Post transplant administration of AMD3100 mobilizes recipient residual HSCs, increasing marrow niche availability. Lethally irradiated C57BL/6 CD45.2 Thy1.2 mice (10.5 Gy) were injected via tail vein with sorted KTLS hematopoietic stem cells (2500 cells/mouse) obtained from C57BL/Ka Thy1.1 donor mice. At 48 hours after transplantation, recipient mice were injected subq with a single dose of AMD3100 at 5 mg/kg or PBS control. Twenty-four hours after this injection, the mice were sacrificed, and the 2 femurs and the spleen were harvested. Recipient-origin stem cells (CD45.2^+^ KLS cells) and donor derived stem cells (CD45.1^+^ KLS cells) in the spleen and in the marrow were determined. Marrow CFUs-GM and Bursting forming units of erythrocytes (BFUs-E) were measured using Methocult® GF 3434 (1×10^5^ cells/dish, triplicate dishes per individual mouse). Percent donor stem cell homing efficiency to the spleen was calculated by dividing the absolute number of CD45.1^+^ KTLS stem cells in the recipient's spleen by the number of injected KTLS stem cells. The graphs are representative of 2 separate sets of experiments with 5–10 mice in each group of each experiment. A: total number of recipient CD45.2^+^ KLS stem cells in 2 femurs. B: marrow CFU-GM and BFU-E assay (numbers of donor CFU-GM and BFU-E/3×10^5^ marrow cells). C: total number of recipient stem cells in the spleen. D: % donor stem cell homing efficiency in the spleen. E: Measurement of hematopoietic stem/progenitor cell mobilization after prolonged AMD3100 administration. Transplanted mice that were given AMD3100 at 5 mg/kg body weight or PBS subcutaneously every other day beginning at day +2 for 60 days and naïve normal control mice were injected with AMD3100 subcutaneously at 5 mg/kg body weight or PBS. Blood samples were collected immediately prior to the injection and 4 hours after injection, and colony-forming units and white blood cell counts determined. Colony-forming units were represented as colony numbers/30 µl blood (n = 5 in each group. Open bar: before AMD3100 injection. Filled bar: 4 hours after AMD3100 injection). *: p<0.05. NS: not statistically significant.

We examined the mechanisms by which AMD3100 selectively enhances donor cell engraftment. We performed additional experiments to investigate the effects of post transplant AMD3100 administration on the mobilization of recipient HSCs and the homing of donor HSCs. Lethally irradiated C57BL/6 **CD45.2** Thy1.2 mice (10.5 Gy) were injected via tail vein with sorted KTLS hematopoietic stem cells (2500 cells/mouse) obtained from C57BL/Ka **CD45.1** Thy1.1 donor mice. At 48 hours after transplantation, recipient mice were injected subcutaneously with a single dose of AMD3100 at 5 mg/kg or PBS control. Twenty-four hours after this injection, the mice were sacrificed, 2 femurs and the spleen harvested, and recipient-originated CD45.2^+^ KLS and donor-derived CD45.1^+^ KLS stem cells measured. As shown in **Fig. 5A**, the mice receiving AMD3100 injection had significantly lower number of recipient CD45.2^+^ KLS stem cells in the marrow, indicating that AMD3100 mobilizes the recipient stem cells from the marrow. In our 2 separate sets of experiments in which lethally irradiated C57Bl/6 CD45.2 Thy 1.2 mice received sorted CD45.1^+^ hematopoietic cells (2500 KTLS cells) and 2 femurs harvested at 72 hrs post transplantation, we were not able to measure donor derived CD45.1^+^ KLS cells in the marrow samples using flow cytometry analysis because of the limited number of KTLS cells infused. Interestingly however, we found that AMD3100-treated mice had significantly higher number of marrow derived CFU-GM and BFU-E, indicating enhanced donor marrow engraftment (**Fig. 5B**). Furthermore, although the number of recipient-origin KLS stem cells in the spleen was similar in both groups (**Fig. 5C**), the AMD3100-treated mice had significantly higher number of donor derived CD45.1^+^ KLS stem cells in the spleen (6.4±1.5%) compared to PBS control mice (0.96±0.36%; p<0.01) (**Fig. 5D**). These results suggest that AMD3100 administered shortly after transplantation increases donor stem cell homing efficiency by expanding the available marrow niches. The available niches are likely expanded as a consequence of AMD3100-induced mobilization of recipient's residual bone marrow cells from marrow niches, allowing a competitive advantage for the healthy donor cells.

To determine if prolonged administration of AMD3100 could still acutely mobilize hematopoietic stem/progenitor cells, we measured colony-forming units and white blood cell counts in peripheral blood 4 hours after giving AMD3100 in transplanted mice that had received AMD3100 every other day beginning at day +2 for 60 days. As shown in **Fig. 5E**, AMD3100 given every other day for 60 days mobilized hematopoietic progenitor cells at a level comparable to those in naïve mice receiving the first dose of AMD3100 injection.

### 6. Post transplant administration of AMD3100 increases donor cell division

**Figure 6:**
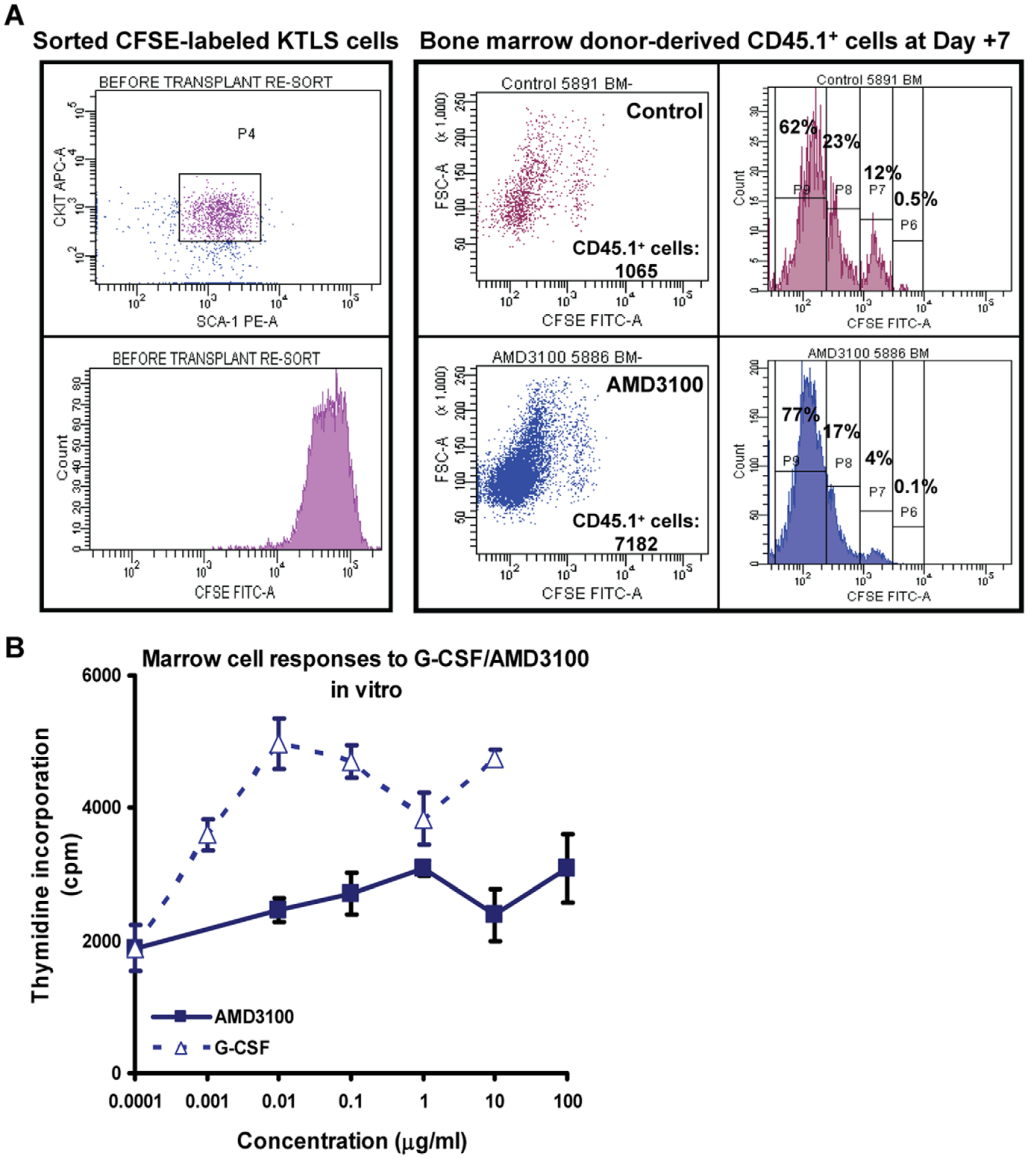
Post transplant administration of AMD3100 increase donor cell division. A: In vivo cell division measurement. CD45.1^+^ KTLS cells were labeled with CFSE and injected into lethally irradiated C57BL/6 CD45.2 Thy1.2 mice (7×10^3^ KTLS cells/mouse). The 2 panels on the left showed the sorted CFSE-labeled KTLS cells prior to injection to recipient mice. The recipient mice were treated with AMD3100 at 5 mg/kg or PBS every other day beginning at day +2. The mice were sacrificed at day +7 and bone marrow cells measured for CFSE intensity (The 4 panels on the right. Top 2 panels: recipient mice treated with PBS control; Lower 2 panels: recipient mice treated with AMD3100). The CFSE intensity was gated on CD45.1^+^ donor derived cell population (n = 5 mice in each group). **B: In vitro marrow cell proliferation responses to AMD3100 or G-CSF.** Marrow cells (5×10^5^/well) were cultured with various concentrations of AMD3100 or recombinant G-CSF, and [^3^H] thymidine incorporation was measured (three separate sets of experiments with triplicates in each experiment).

The proliferative status of HSCs affects their ability to engraft[11], [14], [19] and the speed of hematological recovery[12], [13]. Mobilized HSCs are innately more proliferative[35]. Furthermore, binding of SDF-1 to CXCR4 receptor inhibits the division of primitive HSC cells[14], [19], [20]. To determine whether blocking CXCR4 receptor with AMD3100 could promote *in vivo* cycling of donor HSCs, we injected 5-(and -6)-carboxyfluorescein diacetate succinimidyl ester (CFSE) - labeled donor KTLS cells (**Fig. 6A**) and measured their division status at Day +7 post transplant (**Fig. 6A**). With each cell division, the CFSE is diluted and fluorescence intensity decreases in a step-wise fashion. Compared to control mice, AMD3100- injected mice had reduced percentage of cells in higher intensity peaks (**Fig. 6A** **, lower panels, dot plots and histograms**), indicating more rounds of cell division. Additionally, AMD3100-treated mice had much higher number of donor derived CD45.1^+^ cells compared to PBS-injected mice (**Fig. 6A**: total CD45.1^+^ cells). To rule out direct stimulating effects of AMD3100, we measured ^3^H incorporation *in vitro* in marrow cells co-cultured with various concentrations of AMD3100 or G-CSF. The proliferating effect of AMD3100 on marrow cells was minimal - approximately 100-1000 fold less than that induced by G-CSF (**Fig. 6B**).

### 7. Post transplant administration of AMD3100 maintains hematopoietic stem cell's long-term repopulating capacity

**Figure 7:**
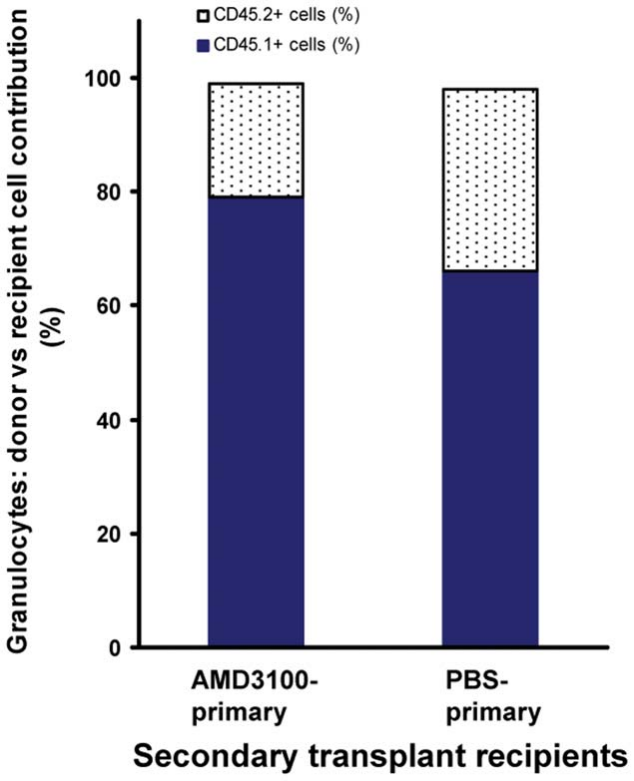
Post transplant administration of AMD3100 maintains HSC's long-term repopulating capacity. Bone marrow cells from transplanted C57BL/6 CD45.2 Thy1.2 recipient mice (i.e., primary transplant recipients) were harvested at day +65, and injected intravenously into lethally irradiated C57BL/6 CD45.2 Thy1.2 mice (i.e., secondary transplant recipients) (one to one matched transplant). The primary transplanted mice received HSCs from donor C57BL/6 CD45.1 mice and were given post transplant administration of AMD3100 at 5 mg/kg subcutaneously every other day from day +2 to +60 (AMD3100-primary) or PBS (PBS-primary). At ∼7 weeks after transplant, the secondary C57BL/6 CD45.2 Thy1.2 transplanted recipient mice were bled and the CD45.1^+^ donor cell contribution in peripheral granulocytes determined. (n = 6 in each group).

The ability of AMD3100 to increase *in vivo* donor cell division and proliferation raises the concern that prolonged post transplant administration of AMD3100 may exhaust stem cell pool. To determine the effects of AMD3100 on HSC self-renewing and long-term repopulating capacities, we performed secondary transplant experiments (**Fig. 7**). We harvested bone marrow cells from transplanted C57BL/6 **CD45.2** Thy1.2 recipient mice at day +65 (i.e., primary transplant recipients). These primary transplanted mice received HSCs from donor C57BL/6 **CD45.1** mice and were given post transplant administration of AMD3100 at 5 mg/kg body weight or PBS subcutaneously every other day from day +2 to +60. The bone marrow cells from primary transplant recipients were then injected intravenously into lethally irradiated C57BL/6 **CD45.2** Thy1.2 mice (i.e., secondary transplant recipients). The whole marrow cells harvested from 1 femur and 2 tibias of one primary mouse were injected into one secondary mouse (one to one matched transplant). At ∼7 weeks after transplant, the secondary C57BL/6 **CD45.2** Thy1.2 transplanted recipient mice were bled and the **CD45.1^+^** donor cell contribution in peripheral granulocytes determined. In the secondary transplant recipients that received bone marrow from AMD3100-treated primary transplant mice, 79±14% blood granulocytes were derived from **CD45.1^+^** donor cells, compared to 66±13% in the mice that received bone marrow from PBS-injected primary transplant mice (**Fig. 7** **;** p>0.05). These data demonstrated that post transplant administration of AMD3100 preserved the long-term repopulating HSC pool.

## Discussion

In our current study, we utilized a specific and reversible CXCR4 antagonist to explore the biological roles of SDF-1/CXCR4 interaction in hematopoiesis and HSC engraftment following HCT. Unlike other chemokines, SDF-1 binds to one single chemokine receptor, CXCR4, which itself is not recognized by any other ligands. CXCR4 knockout mice die in utero and are defective in vascular development, hematopoiesis and cardiogenesis[36]. AMD3100 selectively antagonizes CXCR4 receptor and does not bind to other chemokine receptors. The extreme specificity of AMD3100 makes it a very useful and powerful tool to be used to investigate the role of SDF-1/CXCR4 in HSC engraftment and reconstitution. AMD3100 has a very short half-life, and is rapidly eliminated with an estimated distribution half-life of 0.3 hours and a terminal half-life of 5.3 hours[37]. In our current study, AMD3100 was given every other day. Our results suggest that even a short period of transient CXCR4 blockade could have dramatic impact on animal survival and on HSC engraftment following HCT.

One of our most striking findings of the study was that AMD3100 given after transplant significantly improves survival in lethally irradiated mice transplanted with limited number of hematopoietic stem cells. Interestingly, the recovery of peripheral blood count appears to lag behind the survival benefit (**Fig. 1** **and** **2**). It is not uncommon to see a temporal discrepancy between survival benefit and peripheral blood cell count in transplantation animal models. For example, Chen et al transplanted T cell- depleted bone marrow cells into lethally irradiated allogeneic mice and observed survival benefit at 2 weeks when the peripheral blood counts were still depressed[38]. Most recently, we also observed survival benefit in transplanted mice treated with growth hormone at a time when the peripheral blood count just started to recover[39]. There are 2 possible explanations for this time gap between survival benefit and peripheral blood cell count recovery. First, it is possible that the threshold for peripheral blood count to achieve survival benefit may be low and a slight increase in blood counts may have dramatic impacts on animal survival. Secondly, the peripheral blood count may underestimate the degree and the speed of hematological recovery. In the early recovery phase following transplantation, bone marrow, spleen and other organs are the predominant sites for hematopoiesis.

The differences in blood nucleated cell counts between AMD3100-treated mice and PBS- injected mice became significant at ∼5 weeks after transplantation (**Fig. 2**). The slow peripheral blood hematological recovery and the delayed effects of AMD3100 on hematological reconstitution are likely due to very limited number of KTLS stem cells transplanted in these mice. The mice received approximately 250 KTLS stem cell per mouse in our study. Using a limiting dilution assay, Uchida et al found that engraftment kinetics depended on the number of transplanted HSCs and that the most rapid engraftment occurred when the mice received ≥5000 KTLS cells per mouse[40]. Compared to B cell recovery, AMD3100 had less effect on T cell reconstitution (**Fig. 2**). This discrepancy in the effects of AMD3100 on T and B cell reconstitution results from the different mechanisms of B-cell and T cell regenerations following transplantation[41]. B cell recovery after transplantation recapitulates normal B-cell ontogeny and derives directly from bone marrow lymphoid progenitors. In contrast, T cell reconstitution occurs through both thymic and extrathymic pathways. The contribution of bone marrow lymphoid progenitor cells in T cell reconstitution is limited and highly variable.

Our study suggests that augmented hematological recovery and reduced inflammatory cytokine storm both contribute to the survival benefit observed in AMD3100-treated mice. However, the extent and the relative contribution of each of these 2 mechanisms to animal survival remain to be elucidated. Although our data suggested that hematopoietic stem cells are essential in rescuing these mice from lethal irradiation (**Fig. 3D**), it is possible that AMD3100 enhances hematological recovery by altering the levels of cytokines/chemokines in marrow microenvironment. Recent studies found that many cytokines and chemokines in the niche microenvironment are involved in hematopoiesis following transplantation[5], [6]. Further dissection of the benefit of these 2 mechanisms in survival will require further studies including the use of knockout mice such as MyD88 mice (toll-like receptor knockout mice that fail to elicit a cytokine storm)[42].

Our results demonstrate that the CXCR4 chemokine receptor is involved in transplant-related cytokine storm and subsequent multi-organ toxicity. Blocking CXCR4 with AMD3100 improves animal survival. In addition to HSCs, CXCR4 is also expressed on T cells, B cells, macrophages, dendritic cells, eosinophils, fibroblasts and other inflammatory cells. SDF-1/CXCR4 interaction recruits inflammatory cells to the injury site, exacerbates inflammation and thus plays an important role in the pathogenesis of autoimmune diseases[31], [32], idiopathic lung fibrosis[43], bleomycin-induced lung injury[44], and possibly in acute graft versus host disease[45]. Consistent with the important role of CXCR4 in these disease processes, blocking CXCR4 with AMD3100 attenuates autoimmune responses[31], [32] and improves lung functions[46]. The observation of the decrease in the inflammatory cytokines and improved animal survival by AMD3100 in our current study would suggest its potential use more broadly for any toxic preparatory regimen and for other inflammatory disease processes.

**Figure 8:**
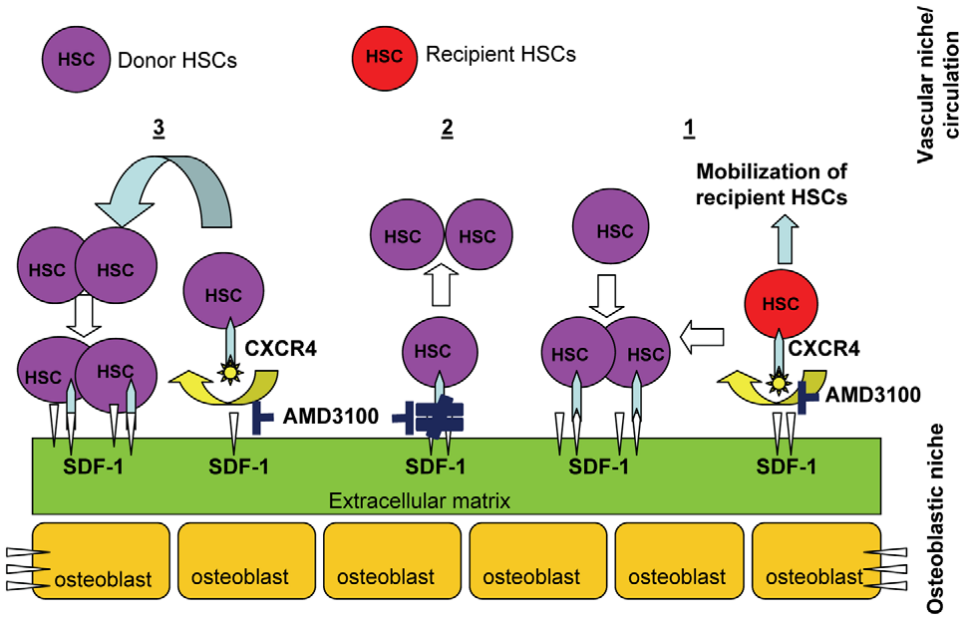
Schematic diagram of AMD3100's mechanisms of action. Post transplant administration of AMD3100 likely has 3 effects. 1. AMD3100 mobilizes residual recipient's radioresistant HSCs (indicated as red circle), thus freeing up more niches for healthy donor HSCs (indicated as purple circles) to engraft. This is supported by **Fig. 5**. 2. AMD3100 relieves the inhibitory effect of SDF-1 on primitive HSCs, allowing for engrafted HSCs to proliferate. 3. AMD3100 mobilizes engrafted donor HSCs and mobilized HSCs are more active and more proliferative. Effects 2 and 3 are supported by **Fig. 6**. Recipient HSCs after irradiation likely have survival disadvantage compared to donor HSCs. The end result is selective engraftment and expansion of donor hematopoietic cells.

Our present studies demonstrate that post transplant administration of AMD3100 selectively enhances donor hematopoietic cell engraftment in a myeloablative setting. Our data suggest that there are three related mechanisms involved in this selective enhancement of donor hematopoietic cell reconstitution by post transplant administration of AMD3100 (**Fig. 8**). The first is that there is mobilization of residual radioresistant host HSCs and thus further opening of hematopoietic niches and a selective advantage for engraftment of the healthy donor HSCs. Secondly, AMD3100 reverses the proliferation-inhibitory effect of SDF-1. SDF-1 arrests the cycling of primitive HSCs[14], [19], [20] and thus blocking the SDF-1/CXCR4 interaction results in proliferation/expansion of primitive HSCs. Thirdly, mobilized HSCs are innately more proliferative than HSCs in the niches[35]. Mobilizing the donor HSCs results in more cell division, further increasing the numbers of donor derived marrow cells. Compared to donor HSCs, recipient residual HSCs likely have survival disadvantage and/or impaired engraftment/proliferation capacity due to the insults from total body irradiation, resulting in selective engraftment and expansion of donor hematopoietic cells.

The number of available niches determines the degree of donor HSC engraftment. In non-ablated mice, AMD3100 can mobilize recipient HSCs, vacate niches and improve donor HSC engraftment[9]. Our data suggest that even in a myeloablative setting, post transplant administration of AMD3100 could further increase the availability of niches and/or keep the marrow niches open when given repeatedly. The restoration and expansion of niches occurs soon after irradiation and is evident at 48 hours post irradiation[47]. Salter et al recently found that infusion of endothelial progenitor cells alone could rescue mice from total body irradiation, suggesting that some host HSCs could survive total body irradiation[48]. Transplant preparative conditioning such as total body irradiation increases the levels of SDF-1 in the marrow. SDF-1 arrests the cycling of very primitive HSCs[14], [19]. In contrast, administration of an SDF-1 antagonist enhances HSC engraftment in vivo[9], [14]. Furthermore, Passegue et al have shown that mobilized HSCs are more proliferative than HSCs in the niches[35]. HSC expansion is crucial to hematological recovery following transplantation[12], [13]. This would also explain the enhancement of donor cell engraftment and recovery seen with repeated administration of AMD3100 following HCT.

We administered AMD3100 at day +2 post transplant and continued the injection every other day. AMD3100 blockade of CXCR4 is reversible and the half-life of AMD3100 is short (terminal half life of ∼5 hours). Thus, it is very likely that the 48 hour interval between AMD3100 injections could allow HSCs to mobilize from “quiescent-storage” niche, enter the cell cycle to expand, and then return to their marrow niches and become quiescent again. This repeated, periodic and pulsed release of HSCs followed by expansion and return to quiescent state may be important in the enhanced hematological recovery observed in our study. Although the effects of persistent and continuous blockade of CXCR4 on hematopoiesis following transplant remain to be elucidated, there is a concern that permanent blockade of CXCR4 might actually impair hematopoiesis. Permanent blockage of CXCR4 may result in the failure of proliferating HSCs to return to “quiescent-storage” niches, leading to increased rate of cell death and impaired hematological recovery.

In our current study, AMD3100 was given every other day for approximately 60 days. We are currently testing various administration regimens including shorter duration of treatment and the use of AMD3100 as part of conditioning regimens. Our preliminary study indicated that shorter duration of treatment such as every other day for 30 days was as effective as 60 day administration (unpublished data).

There were several conflicting articles reporting the effects of SDF-1 and CXCR4 antibody on hematopoiesis after transplant[14], [17], [19], [20], [49], [50]. For example, Peled et al reported reduced homing and impaired engraftment with treatment of graft cells with CXCR4-blocking antibody[17], while Plett et al suggested increased repopulating potential of treated CD34^+^ cells[50]. One study suggested that in vivo administration of SDF-1 was accompanied by enhanced transplantability of human CD34^+^ cells in NOD/SCID mice[20], whereas others reported increased engraftment with SDF-1 antagonist[9], [14]. These discrepancies are likely related to the differences in transplant models, the proliferative status of the grafts, the specificities of CXCR4 antibodies/antagonists and even the concentration of SDF-1. SDF-1 exhibits “concentration-dependent bifunctional effects”, i.e., SDF-1 can attract or repel cells at different concentrations[50]. Furthermore, AMD3100 may act as CXCR4 partial agonist at high concentration[51]. Our findings are similar to those reported by Plett et al[50], Chen et al[9], Bowie et al[14], and Abraham et al[49] using a novel CXCR4 antagonist (4F-benzoyl-TN14003). Abraham et al recently reported enhanced recovery of marrow after transplantation with the CXCR4 antagonist 4F-benzoyl-TN14003[49].

The overall effects of the blockage of CXCR4/SDF-1 with AMD3100 on hematopoiesis are outlined in **Fig. 8**. Additionally, AMD3100 could affect molecular components of niche microenvironment such as cytokines, chemokines, neurotransmitters, and oxidative status that are crucial to the homing, proliferation and mobilization of HSCs. Our results indicate that AMD3100 promotes recovery of all donor cell lineages, including myeloid cells, T and B lymphocytes, erythrocytes and platelets. The data suggest that AMD3100 enhances engraftment of multipotent primitive HSCs. Furthermore, as shown in **Fig. 2** and **4**, the total blood counts and marrow KLS stem cells in AMD3100-treated mice at day +65 were significantly higher than those in PBS-injected control mice. Moreover, using secondary transplant model, we found that prolonged post transplant administration of AMD3100 did not adversely affect the long-term engraftment of primitive HSCs (**Fig. 7**).

Our current study provides important insights into the roles of SDF-1/CXCR4 interaction in transplant-related mortality and in hematopoiesis following HCT. Our study suggests that blocking SDF-1/CXCR4 interaction with CXCR4 antagonist has many important ramifications not only in clinical HSC transplantation, but also potentially in other clinical settings that involve exaggerated inflammatory responses. Since AMD3100 is FDA approved for mobilization of HSCs, the translational potential of this agent is significant. Its use to enhance engraftment and decrease the toxicity would be especially important in clinical setting such as cord blood transplantation where engraftment remains a problem. AMD3100 can also be used routinely in any myeloablative allogeneic transplantation to ensure robust donor cell engraftment. Our studies indicate that post transplant administration of AMD3100 is a new approach for enhancing hematological recovery and reducing transplant-related mortality in HCT.

## Methods

### Animals

Eight to twelve week old C57BL/6, CD45.2, Thy1.2 (H2^b^, termed “CD45.2 mice”) mice were purchased from the Jackson Laboratories (Bar Harbor, ME). C57BL/Ka, CD45.1, Thy1.1 mice (H2^b^, termed ^“^CD45.1 mice”) were bred and maintained in our specific pathogen-free animal facility. Studies were performed in accordance with Duke University Institutional Animal Care and Use Committee-approved procedures.

### Antibodies and reagents

AMD3100 used in our first 2 sets of *in vivo* experiments was purchased from Sigma (St Louis, MO). AMD3100 (Plerixafor, Mozobil™, Genzyme Corp.) provided by Genzyme Corp under a research agreement was used in our third set of *in vivo* experiment and in all other studies, including stem cell homing studies, cytokine studies and *in vivo* proliferation experiments. Antibodies for phenotypic analyses included: R-phycoerythrin (PE)-conjugated anti-CD45.1 (clone A20); FITC- conjugated anti-CD45.2 (clone 104); APC-Cy7-labeled anti-CD45.2; APC-conjugated anti-CD3 (clone 145-2C11); APC-Cy7-labeled anti mouse CD4 (L3T4, clone GK1.5); PE-Cy7 labeled anti mouse CD8a (Ly-2, clone 53–6.7); Biotin conjugated anti mouse Gr-1 (clone RB6-8C5), and APC conjugated anti mouse CD117 (c-kit, clone 2B8). All of these antibodies were from BD Pharmingen (San Diego, CA). Tricolor-conjugated anti mouse CD45R (B220) was from Caltag (South San Francisco, CA); and PE-Cy7 conjugated anti mouse Sca-1 (clone D7) was from eBiosciences (San Diego, CA). Antibodies used for purification of hematopoietic stem cells are noted below.

### Murine hematopoietic stem cell transplantation

Donor mouse c-Kit^+^Thy1.1^low^Lin^−/low^Sca-1^+^ hematopietic stem cells (KTLS cells) were obtained from CD45.1 mice as described previously[52]. Briefly, marrow cells prepared from the CD45.1 mice were first depleted of red blood cells using ACK solution (0.15 M ammonium and 0.01 M potassium carbonate). Red cell-depleted bone marrow cells were then enriched for c-Kit^+^ cells by positive selection using purified anti-c-Kit antibody (clone ACK45; BD Pharmingen) followed by incubation with goat anti-rat magnetic beads from Miltenyi Biotec. C-Kit-enriched bone marrow cells were subsequently stained with a cocktail of primary monoclonal antibodies consisting of biotin-conjugated anti-CD3 (clone 145-2C11), anti-CD4 (clone RM4-5), anti-CD5 (clone 53–7.3), anti-CD8a (clone 53–6.7), anti-CD11b (clone M1/70), anti-B220 (clone RA3-6B2), anti-Gr-1 (clone RB6-8C5), and anti-TER-119 (clone TER-119); FITC-conjugated anti-Thy1.1 (clone OX-7); PE-conjugated anti-Sca-1 (clone E13-161.7); and allophycocyanin-conjugated anti-c-Kit (clone 2B8); followed by secondary staining with streptavidin-Cy-Chrome (all purchased from BD Pharmingen). Propidium iodide (1 µg/mL; Sigma, St Louis, MO) was added to the cell suspension to exclude dead cells before cell sorting. The stained samples were sorted for c-Kit^+^Thy1.1^low^Lin^−/low^Sca-1^+^ hematopoietic stem cells (KTLS) cells using a dual laser fluorescence activated cell sorter (FACS) Vantage SE (Becton Dickinson, San Jose, CA).

The recipient CD45.2 mice were given total body irradiation using a cesium irradiator. The mice received 10.5 Gy irradiation in the first 2 sets of experiments and 9.5 Gy in the third set of experiment. Within 4 hours after irradiation, the CD45.2 mice were injected via tail vein with the sorted KTLS hematopoietic stem cells (250 KTLS cells per mouse).

### AMD3100 administration

The transplanted mice were injected subcutaneously with PBS mock control or AMD3100 at 5 mg/Kg body weight in a volume of 100 µl every Monday, Wednesday and Friday beginning at day +2 post transplant until day +56. Animal survival was monitored daily.

### Measurement of hematological recovery

To determine peripheral blood hematological recovery and to quantify various cell subsets, blood samples were obtained using heparinized capillary tubes at the indicated time points. Fifty micro-liters of blood were stained with monoclonal antibodies for 15 minutes at room temperature. The stained whole-blood samples were then processed in BD FACS™ Lysing solution to lyse red blood cells. Flow-Count fluorospheres (50 µL; Beckman-Coulter) were added before flow cytometric analysis. Stained cells were analyzed using BD FACSCanto™ (BD Biosciences, San Jose, CA). The absolute counts were calculated using the following formula: Absolute count (cells/µL blood)  =  (Total number of cells counted/Total number of fluorospheres counted) x Flow-Count fluorosphere concentration. Hemoglobin concentration and platelet counts were measured using a Hemavet® counter (Drew Scientific, Inc, Dallas, Texas) as per manufacturer's instruction.

To determine marrow and spleen hematopoietic stem/progenitor cell recovery, the transplanted recipient mice were sacrificed at day +65, and 2 femurs, 2 tibias and the spleen were harvested. Several analyses were performed including histological examination, nucleated marrow cell counts, colony forming unit assay (CFUs), and stem cell quantification. One femur and one tibia were used for histological examination. The femur and tibia were fixed in 10% buffered neutral formalin (VWR International, West Chester, PA). Bone was decalcified, stained with H&E, and examined by microscopy. The other femur and tibia marrow cavities were flushed, and marrow cells were resuspended in 4 ml of RPMI1640 containing 10% fetal calf sera. The total nucleated cells of the femur and the tibia were counted using the Hemavet® counter. To determine the frequency of colony forming units-granulocytes and megakaryocytes (CFUs-GM), marrow cells from individual mice were plated in Methocult® GF 3434 (1×10^5^ cells/dish, triplicate dishes per individual mouse) as per manufacturer's instruction (StemCell Technology, Vancouver, Canada). The number of CFUs-GM was counted at day 12. To determine the numbers of donor- derived CD45.1^+^ c-kit^+^ Lin^−^ Sca-1^+^ (KLS) hematopoietic stem cells and recipient-origin CD45.2^+^ KLS cells, the marrow cell suspension was stained with PE-conjugated CD45.1 antibody, FITC-conjugated CD45.2, APC-labeled c-Kit antibody, PE-Cy5 conjugated Sca-1 and biotin-conjugated anti-CD3 (clone 145-2C11), anti-CD4 (clone RM4-5), anti-CD5 (clone 53-7.3), anti-CD8a (clone 53-6.7), anti-CD11b (clone M1/70), anti-B220 (clone RA3-6B2), anti-Gr-1 (clone RB6-8C5), and anti-TER-119 (clone TER-119); followed by secondary staining with streptavidin-Cy-Chrome. The absolute KLS stem cell number in one femur and one tibia was calculated multiplying total number of nucleated marrow cells with % KLS cells on flow cytometry analysis. The absolute cell numbers of recipient-origin stem cells (CD45.2^+^ KLS cells) and donor derived stem cells (CD45.1^+^ KLS cells) in the spleen were similarly determined.

### Cytokines/chemokines measurement

Lethally irradiated CD45.2 mice were transplanted with KTLS cells and treated with PBS or AMD3100 as described above. At day +7 post transplantation, the mice were bled and plasma prepared. The cytokines/chemokines (pg/ml) was measured using Bioplex-mouse 23 Plex Cytokines Kit as per manufacturer's instruction (Bio-Rad Laboratories, Hercules, CA). These cytokines/chemokines are IL-1α, IL-1β, IL-2, IL-3, IL-4, IL-5, IL-6, IL-9, IL-10, IL-12(p40), IL-12(p70), IL-13, IL-17, Eotaxin, G-CSF, GM-CSF, IFN-λ, keratinocyte chemoattractant (KC), monocyte chemoattractant protein-1 (MCP-1), MIP-1α, MIP-1β, TNF-α, and RANTES.

### Measurement of colony forming unit of spleen at day +9

Lethally irradiated CD45.2 mice (10.5 Gy) were injected with whole marrow cells from CD45.1 mice (1×10^5^ marrow cells per mouse). The recipient mice were injected subcutaneously with PBS buffer or AMD3100 at 5 mg/kg every other day beginning at day +2. At day +9, the mice were sacrificed and the spleen was harvested and fixed in Bouin's solution. The colony forming units of spleen (CFUs-spleen) were counted.

### *In vivo* stem cell mobilization and homing studies shortly after transplantation

Lethally irradiated C57BL/6 CD45.2 Thy1.2 mice (10.5 Gy) were injected via tail vein with sorted KTLS hematopoietic stem cells (2500 cells/mouse) obtained from C57BL/Ka Thy1.1 donor mice. At 48 hours after transplantation, recipient mice were injected subq with a single dose of AMD3100 at 5 mg/kg or PBS control. Twenty-four hours after this injection, the mice were sacrificed, and the 2 femurs and the spleen were harvested. The total nucleated cells in the 2 femurs and in the spleen were measured using the Hemavet® counter. The absolute cell numbers of recipient-origin stem cells (CD45.2^+^ KLS cells) and donor derived stem cells (CD45.1^+^ KLS cells) in the spleen and in the marrow were determined, as described above. Marrow CFUs-GM and Bursting forming units of erythrocytes (BFUs-E) were measured using Methocult® GF 3434 (1×10^5^ cells/dish, triplicate dishes per individual mouse) as per manufacturer's instruction (StemCell Technology, Vancouver, Canada). Percent donor stem cell homing efficiency to the spleen was calculated by dividing the absolute number of CD45.1^+^ KTLS stem cells in the recipient's spleen by the number of injected KTLS stem cells. To determine the mobilization of recipient's residual marrow stem cells from marrow, we measured the recipient-origin stem cells (CD45.2^+^ KLS cells) in the marrow by flow cytometry as described above.

### Measurement of hematopoietic stem/progenitor cell mobilization after prolonged administration of AMD3100

Transplanted mice that were given AMD3100 at 5 mg/kg body weight or PBS subcutaneously every other day beginning at day +2 for 60 days were used in this study. Additionally, naïve mice that were not transplanted and had not received AMD3100 prior to the study were used as positive control. The mice were given AMD3100 at 5 mg/kg body weight or PBS. Blood samples were collected immediately prior to the injection and 4 hours after injection, and colony-forming units and white blood cell counts determined. Colony-forming units were represented as colony numbers/30 µl blood.

### *In vivo* cell division analysis

KTLS cells obtained from donor CD45.1 mice were labeled with CFSE [5-(and -6)-carboxyfluorescein diacetate succinimidyl ester, Molecular Probes, Eugene, OR] at a final concentration of 5 µM and incubated at 37°C for 10 min[53]. After the incubation period, the cells were immediately washed three times in cold RPMI/10% FCS. The CFSE-labeled KTLS cells were re-sorted by flow cytometry to obtain a pure population of cells that were CFSE^+^ c-Kit^+^ Thy1^low^ Lin^−^ Sca-1^+^. The CFSE-labeled KTLS cells were then injected into 10.5 Gy irradiated CD45.2 recipient mice (7,000 KTLS cells/mouse). Beginning at day +2, the recipient mice were injected subcutaneously with AMD3100 at 5 mg/kg or PBS control every other day. The mice were sacrificed at day +7, and CFSE positive cells were measured by flow cytometry. CD45.1^+^ cells were analyzed for CSFE content.

### *In vitro* proliferation assay

Marrow cells from normal mice (5×10^5^/well) were cultured with various concentrations of AMD3100 or recombinant G-CSF (Neupogen®, Amgen Inc, Thousand Oaks, CA) at a volume of 200 µl in 96 well plates. Forty-eight hours later, 0.5 µCi of [^3^H] thymidine was added, cells harvested at 18 hrs and the incorporated [^3^H] thymidine detected by using Liquid Scintillation & Luminescence Counter (PerkinElmer LAS, Shelton, CT).

### Secondary transplantation experiment

Bone marrow cells from transplanted C57BL/6 **CD45.2** Thy1.2 recipient mice (i.e., primary transplant recipients) were harvested at day +65, and injected intravenously into lethally irradiated C57BL/6 **CD45.2** Thy1.2 mice (i.e., secondary transplant recipients) (one to one matched transplant). The **primary** transplanted mice received HSCs from donor C57BL/6 **CD45.1** mice and were given post transplant administration of AMD3100 at 5 mg/kg or PBS subcutaneously every other day from day +2 to +60. At ∼7 weeks after transplant, the **secondary** C57BL/6 **CD45.2** Thy1.2 transplanted recipient mice were bled and the **CD45.1^+^** donor cell contribution in peripheral granulocytes determined.

### Statistical analysis

All values were reported as mean ± SEM of multiple measurements. Statistical comparisons between AMD3100-treated mice and PBS-injected mock mice were done using the Student *t* test. Statistical significance was defined as p<0.05.
